# Immune cell-derived cytokines contribute to obesity-related inflammation, fibrogenesis and metabolic deregulation in human adipose tissue

**DOI:** 10.1038/s41598-017-02660-w

**Published:** 2017-06-07

**Authors:** Charles Caër, Christine Rouault, Tiphaine Le Roy, Christine Poitou, Judith Aron-Wisnewsky, Adriana Torcivia, Jean-Christophe Bichet, Karine Clément, Michèle Guerre-Millo, Sébastien André

**Affiliations:** 1 INSERM, UMR_S 1166, Team 6 Nutriomics, F-75013 Paris, France; 2 Sorbonne Universités, UPMC Université Paris 06, UMR_S 1166, F-75005 Paris, France; 3 Institute of Cardiometabolism and Nutrition, ICAN, Pitié-Salpêtrière Hospital, Assistance Publique Hôpitaux de Paris, F-75013 Paris, France; 4 Assistance Publique Hôpitaux de Paris, Pitié-Salpêtrière Hospital, Nutrition and Endocrinology Department, F-75013 Paris, France; 5 Assistance Publique Hôpitaux de Paris, Pitié-Salpêtrière Hospital, Hepato-biliary and Digestive Surgery Department, F-75013 Paris, France; 6 Assistance Publique Hôpitaux de Paris, Pitié-Salpêtrière Hospital, Plastic surgery and Mammary Cancer Department, F-75013 Paris, France

## Abstract

Adipose tissue contains a variety of immune cells, which vary in abundance and phenotype with obesity. The contribution of immune cell-derived factors to inflammatory, fibrotic and metabolic alterations in adipose tissue is not well established in human obesity. Human primary adipose tissue cells, including pre-adipocytes, endothelial cells and mature adipocytes, were used to investigate deregulation of cell- and pathway-specific gene profiles. Among factors known to alter adipose tissue biology, we focus on inflammatory (IL-1β and IL-17) and pro-fibrotic (TGF-β1) factors. rIL-1β and rIL-17 induced concordant pro-inflammatory transcriptional programs in pre-adipocytes and endothelial cells, with a markedly more potent effect of IL-1β than IL-17. None of these cytokines had significant effect on fibrogenesis-related gene expression, contrasting with rTGF-β1-induced up-regulation of extracellular matrix components and pro-fibrotic factors. In mature adipocytes, all three factors promoted down-regulation of genes functionally involved in lipid storage and release. IL-1β and IL-17 impacted adipocyte metabolic genes in relation with their respective pro-inflammatory capacity, while the effect of TGF-β1 occurred in face of an anti-inflammatory signature. These data revealed that IL-1β and IL-17 had virtually no effect on pro-fibrotic alterations but promote inflammation and metabolic dysfunction in human adipose tissue, with a prominent role for IL-1β.

## Introduction

In obesity, the adipose tissue is a site of immune cell accumulation, which maintains a state of chronic low-grade inflammation in absence of infection. Cells from both the innate and adaptive arms of the immune system are detected in distinct abundance and phenotype (reviewed in ref. 1). Adipose tissue colonization by pro-inflammatory macrophages is a hallmark of obesity^2–5^. Numerous *in vitro* studies have stressed the role of macrophages as a prominent source of bioactive molecules with a potential to induce inflammatory, fibrotic or insulin resistant states in adipose tissue non-immune cell types^6–12^. Several macrophage-derived factors, including TNF-α^6, 8, 9^ and IL-β^11, 12^ have been implicated to mediate the inflammatory and catabolic effects of macrophages on adipose cells. Rodent and human studies showing that anti-TNF-α^13, 14^ or anti-IL-1β^15–17^ immunotherapy improved glycemic status support implication of these cytokines in linking adipose macrophage accumulation to metabolic derangement. IL-6, another cytokine prominently released by pro-inflammatory macrophages, is known to alter insulin signaling and promote inflammation in murine adipocytes^18, 19^. In humans, opposite change in adipose tissue IL-6 content and whole body insulin sensitivity occurs upon body weight variation^20, 21^. Recently, TGF-β family members were shown to contribute to the pro-fibrogenic effect of macrophage conditioned medium on adipose tissue endothelial or progenitor cells^10, 22^. Collectively, these observations emphasize the pathological relevance of macrophage-derived cytokines to impact adipose tissue biology with deleterious systemic consequences during obesity.

We and others recently revealed that, besides macrophages, Th17 cells, a subset of CD4^+^ T lymphocytes accumulate in adipose tissue in relation with increased fat mass and altered subjects’ glycemic status^23–25^. Th17 cells produce specific cytokines, including IL-17 and IL-22. In co-culture experiments using human adipose tissue-derived primary cells, we showed that IL-22 increased the release of IL-1β by macrophages, while IL-1β enhanced the production of Th17 cytokines by autologous CD4^+^ T cells. This pro-inflammatory paracrine loop was amplified in type 2 diabetic subjects and attenuated after bariatric surgery-induced weight loss, congruent with variations in blood glycemic variables^25^. Thus, reciprocal amplification of IL-1β and Th17 cytokines in adipose tissue appeared critical to sustain local inflammation and systemic glycemic deterioration in human obesity.

In the present study, we hypothesized that adipose tissue non-immune cells might also be targeted by immune cell-derived cytokines, thus contributing to metabolic deterioration. An effect of IL-1β to promote inflammation and insulin resistance in cultured adipose cells has been previously reported^12, 26, 27^. However, less is known on the capacity of Th17 cytokines to affect adipose tissue biology, particularly in humans. In mouse studies, adipocyte specific overexpression of IL-22 increased the production of inflammatory cytokines in adipose tissue, but did not alter mice metabolic phenotype^28^. In another study, however, pharmacological administration of IL-22 dampened adipose tissue inflammation and restored insulin sensitivity in obese mice^29^, suggesting a positive effect of the cytokine at the systemic level. IL-17 impaired insulin action in murine adipocytes, although mice lacking IL-17 were not protected against diet-induced obesity and insulin resistance^30^. Finally, at odds with accumulation of Th17 cells in human obesity, IL-17 deficient mice displayed increased adiposity^30^ supported by IL-17 anti-adipogenic effect demonstrated *in vitro* ^31^. In sum, these mouse observations did not unambiguously establish implication of Th17-related cytokines to drive obesity-induced deterioration of adipose tissue biology.

The aim of the present study was to explore the contribution of immune cell-derived factors to alter adipose tissue cell biology in humans. This was investigated in primary cells, including pre-adipocytes, CD31^+^ endothelial cells and mature adipocytes, which are routinely used in our team to address the bases of human adipose tissue alterations^9, 10, 32–34^. Here, we ought to test the capacity of IL-1β, Th17-related cytokines and TGF-β1 to induce inflammation and fibrogenesis and to alter adipocyte metabolic capacity.

## Results

### Obese adipose tissue microenvironment promotes IL-17 release

**Figure 1:**
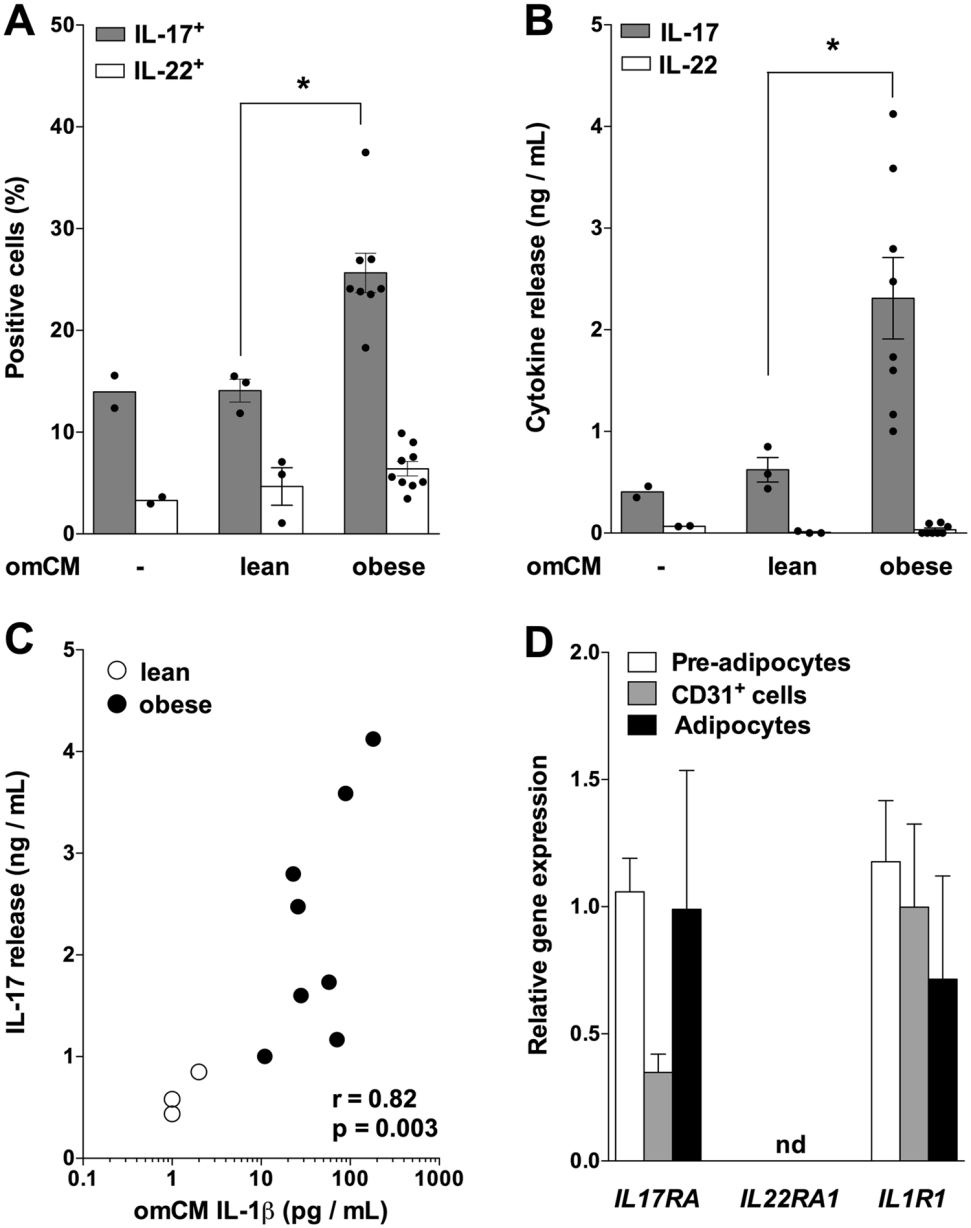
Obese omental adipose tissue microenvironment promotes IL-17 release. (**A**) IL-17^+^ and IL-22^+^ cells were quantified by FACS analysis of blood-derived memory CD4^+^ T cells cultured for 7 days without or with conditioned medium of omental adipose tissue (omCM) from lean (n = 3) or obese (n = 8) subjects. *P < 0.05. (**B**) IL-17 and IL-22 concentrations were determined by Luminex in blood-derived memory CD4^+^ T cells culture medium. *P < 0.05. (**C**) Correlation analysis between IL-17 concentration in blood CD4^+^ T cells culture medium and IL-1β concentration in omCM. The correlation coefficient (r) and p value were obtained by Spearman’s test. (D) IL17RA, IL22RA1 and IL1R1 mRNA were determined in pre-adipocytes (n = 7), CD31^+^ endothelial cells (n = 6) and adipocytes (n = 4) obtained from lipoaspirate adipose tissue samples. Data are shown as mean ± SEM. nd: not detected.

To substantiate the role of adipose tissue microenvironment on T cell phenotype, we obtained conditioned media from omental adipose tissue (omCM) in lean and obese subjects. Marked and concordant increases in IL-17^+^ cells and IL-17 release by blood memory CD4^+^ T cells were found in response to omCM from obese subjects (Fig. 1A,B). In this cell model, however, IL-22^+^ cells and IL-22 release remained low or undetectable. Notably, the amplitude of IL-17 production by CD4^+^ T cells was positively correlated with IL-1β concentrations in omCM (Fig. 1C), supporting the role of adipose tissue IL-1β to trigger IL-17 release by Th17 polarized T cells. This does not preclude that other cytokines present in adipose tissue microenvironment contribute to IL-17 release by T cells in human obesity (Supplementary Table S1).

As a prerequisite to assess and confront the effects of Th17 cytokines and IL-1β on adipose tissue non-immune cells, we determined expression of their cognate receptors in pre-adipocytes, CD31^+^ endothelial cells and mature adipocytes. While the signaling receptors *IL17RA* and *IL1R1* were readily expressed in the three cell types, *IL22RA1* mRNA was not detected (Fig. 1D). Therefore, the next series of experiments was focused on comparing the effects of IL-17 and IL-1β in the three primary human cell types.

### Pro-inflammatory signature in human adipose tissue non-immune cells

Pre-adipocytes were screened by PCR array for change in the expression of 84 genes related to inflammation and extracellular matrix remodeling in response to human rIL17 or rIL-1β. Both cytokines induced an inflammatory signature, but of different nature (Table 1). According to a 2-fold induction threshold, 41 genes were induced by rIL-1β, while only 8 genes of the list were up-regulated by rIL-17. Moreover, the amplitude of rIL-1β stimulatory effect was markedly higher than that of rIL-17, when considering the 8 genes up-regulated by both cytokines.

**Table 1:** PCR array analysis of 84 genes related to fibrosis and inflammation in primary human pre-adipocytes.

Symbol	Refseq	Description	rIL-1β	rIL-17
**MMP1**	NM_002421	Matrix metallopeptidase 1 (interstitial collagenase)	924.2	4.8
**MMP3**	NM_002422	Matrix metallopeptidase 3 (stromelysin 1, progelatinase)	882.8	2.7
**IL1B**	NM_000576	Interleukin 1, beta	412.7	6.9
**CCL2**	NM_002982	Chemokine (C-C motif) ligand 2	137.8	6.1
**MMP8**	NM_002424	Matrix metallopeptidase 8 (neutrophil collagenase)	98.8	6.4
CCL3	NM_002983	Chemokine (C-C motif) ligand 3	48.2	−1.2
CXCR4	NM_003467	Chemokine (C-X-C motif) receptor 4	42.1	−2.7
ITGA2	NM_002203	Integrin, alpha 2 (CD49B, alpha 2 subunit of VLA-2 receptor)	35.1	−1.9
IL1A	NM_000575	Interleukin 1, alpha	26.1	−1.1
**CEBPB**	NM_005194	CCAAT/enhancer binding protein (C/EBP), beta	24.9	2.3
SERPINA1	NM_000295	Serpin peptidase inhibitor, clade A (alpha-1 antiproteinase, antitrypsin), member 1	16.3	1.9
**SERPINE1**	NM_000602	Serpin peptidase inhibitor, clade E (nexin, plasminogen activator inhibitor type 1), member 1	11.5	2.8
MMP14	NM_004995	Matrix metallopeptidase 14 (membrane-inserted)	6.5	1.9
LTBP1	NM_000627	Latent transforming growth factor beta binding protein 1	6.4	−1.3
JUN	NM_002228	Jun proto-oncogene	5.7	1.5
TNF	NM_000594	Tumor necrosis factor	5.4	−3.9
MMP9	NM_004994	Matrix metallopeptidase 9 (gelatinase B, 92 kDa gelatinase, 92 kDa type IV collagenase)	4.7	−1.1
**GREM1**	NM_013372	Gremlin 1	4.7	2.7
TGFBR1	NM_004612	Transforming growth factor, beta receptor 1	4.1	1.1
ITGA3	NM_002204	Integrin, alpha 3 (antigen CD49C, alpha 3 subunit of VLA-3 receptor)	4,0	−1.2
TGFB2	NM_003238	Transforming growth factor, beta 2	3.7	1.3
TGIF1	NM_003244	TGFB-induced factor homeobox 1	3.6	1.1
NFKB1	NM_003998	Nuclear factor of kappa light polypeptide gene enhancer in B-cells 1	3.6	1.1
SMAD7	NM_005904	SMAD family member 7	3.5	−2,0
IL13RA2	NM_000640	Interleukin 13 receptor, alpha 2	3.5	−1.1
ITGB1	NM_002211	Integrin, beta 1 (fibronectin receptor, beta polypeptide, antigen CD29 includes MDF2, MSK12)	3.5	−1,0
PLAU	NM_002658	Plasminogen activator, urokinase	3.4	1.1
ITGA1	NM_181501	Integrin, alpha 1	3.4	−1.1
COL3A1	NM_000090	Collagen, type III, alpha 1	3.4	1.5
TGFB1	NM_000660	Transforming growth factor, beta 1	3.3	1.9
TIMP1	NM_003254	TIMP metallopeptidase inhibitor 1	3.1	−1.6
VEGFA	NM_003376	Vascular endothelial growth factor A	3,0	−1.9
THBS1	NM_003246	Thrombospondin 1	2.9	1.5
THBS2	NM_003247	Thrombospondin 2	2.8	−1,0
TIMP4	NM_003256	TIMP metallopeptidase inhibitor 4	2.7	1.1
STAT1	NM_007315	Signal transducer and activator of transcription 1, 91 kDa	2.5	1.3
SMAD3	NM_005902	SMAD family member 3	2.3	1,0
MMP2	NM_004530	Matrix metallopeptidase 2 (gelatinase A, 72 kDa gelatinase, 72 kDa type IV collagenase)	2.2	1.1
MYC	NM_002467	V-myc myelocytomatosis viral oncogene homolog (avian)	2.1	−1.5
PDGFA	NM_002607	Platelet-derived growth factor alpha polypeptide	2.1	−1.1
HGF	NM_000601	Hepatocyte growth factor (hepapoietin A; scatter factor)	2.0	−3.6

Cells were treated or not by recombinant rIL-1β (10 ng/mL) or rIL-17 (10 ng/mL). The data show fold changes over untreated control cells for 41 genes up-regulated by rIL-1β with a 2-fold threshold. Bold labeling identifies 8 genes up-regulated by rIL-17 in the same culture.

**Figure 2:**
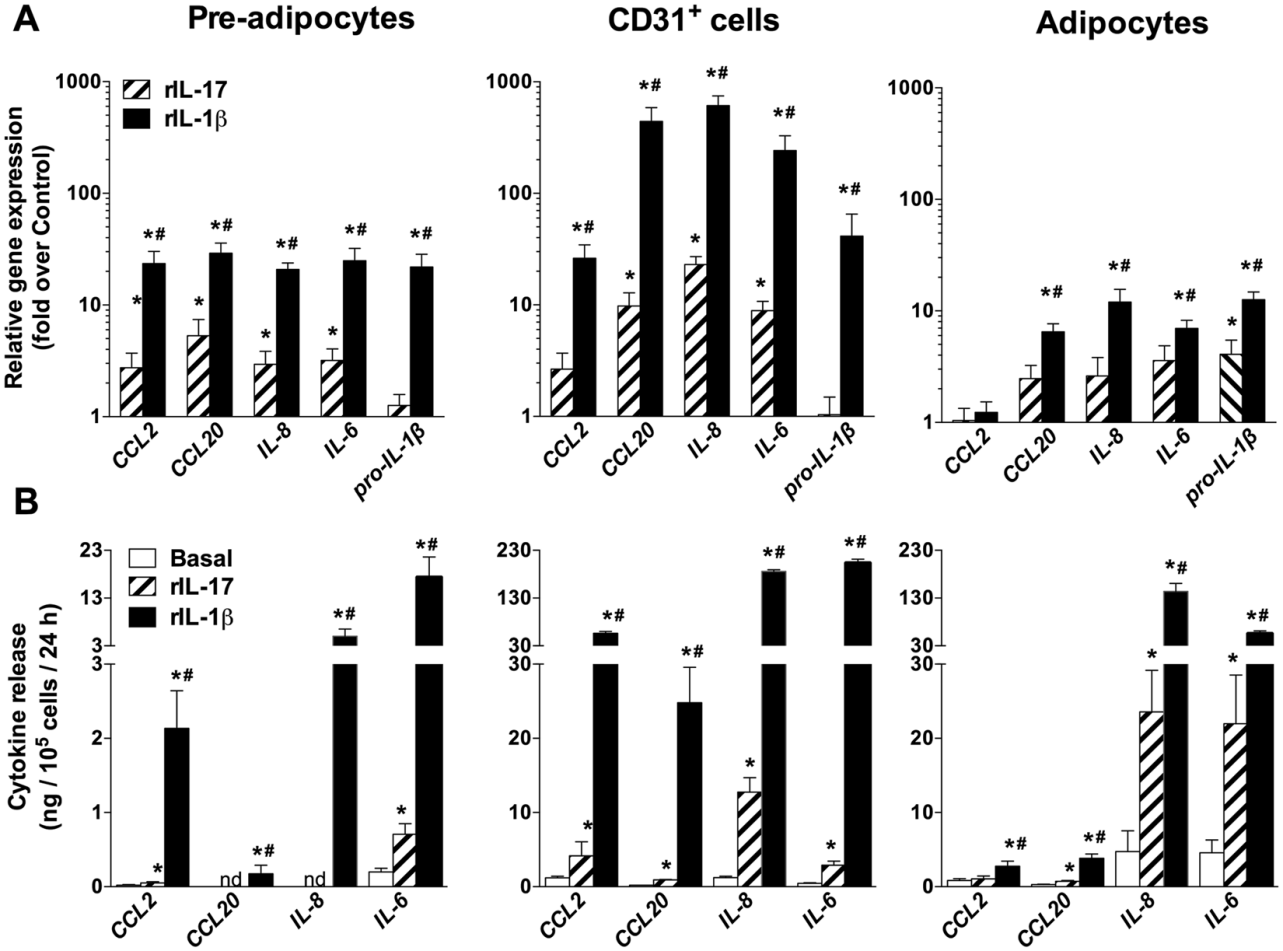
Pro-inflammatory signature in human adipose tissue non-immune cells. (**A**) *CCL2*, *CCL20*, *IL-8*, *IL-6* and *pro-IL-1β* mRNA were determined by RT-qPCR in pre-adipocytes (n = 10) and CD31^+^ endothelial cells (n = 6) cultured for 96 h and by QuantiGene Plex in adipocytes 3D cultured for 72 h (n = 10) without or with rIL-17 (10 ng/mL) or rIL-1β (10 ng/mL). *P < 0.05 *versus* untreated cells. ^#^ *P* < 0.05 *versus* rIL-17 treated cells. (**B**) Concentrations of CCL2, CCL20, IL-8 and IL-6 were quantified by Luminex technology in culture media of pre-adipocytes (n = 6), CD31^+^ endothelial cells (n = 6) and adipocytes (n = 7) in untreated conditions (white bars) and in response to rIL-17 (stripped bars) or rIL-1β (black bars). *P < 0.05 *versus* untreated cells. #*P* < 0.05 *versus* rIL-17 treated cells. Data are shown as mean ± SEM.

We extended this exploration to pre-adipocytes, CD31^+^ endothelial cells and mature adipocytes, focusing on a set of inflammation-related genes (*CCL2, CCL20, IL-8, IL-6* and pro*-IL-1β*) selected among genes known to be up-regulated in adipose tissue with human obesity. With the exception of *CCL2* in adipocytes, these genes were markedly up-regulated by rIL-1β and, to a lesser extent, by rIL-17 (Fig. 2A). Of note, potent IL-1β self-induction was observed, while up-regulation of *pro-IL1β* gene by rIL-17 occurred only in adipocytes.

The release of corresponding proteins demonstrated cell- and cytokine-specific patterns (Fig. 2B). In pre-adipocytes, low or even undetectable levels of CCL2, CCL20, IL-8 and IL-6 proteins were observed both in basal conditions and in response to recombinant cytokines. In the other cell types, rIL-1β and rIL-17 exerted a stimulatory effect prominently on IL-8 and IL-6 release. In line with gene expression data (Fig. 2A), the stimulatory effect of rIL-1β was always much larger than that of rIL-17. Small amounts of mature IL-1β (9.3 ± 1.7 pg/10^5^ cells/24 h) were released by adipocytes and increased 2-fold in response to rIL-17. In presence of both cytokines combined, evoked inflammatory responses were of similar amplitude as that induced by rIL-1β alone, both at gene and protein levels (data not shown).

### Cytokine-specific effect on extracellular matrix and fibrotic genes

**Figure 3:**
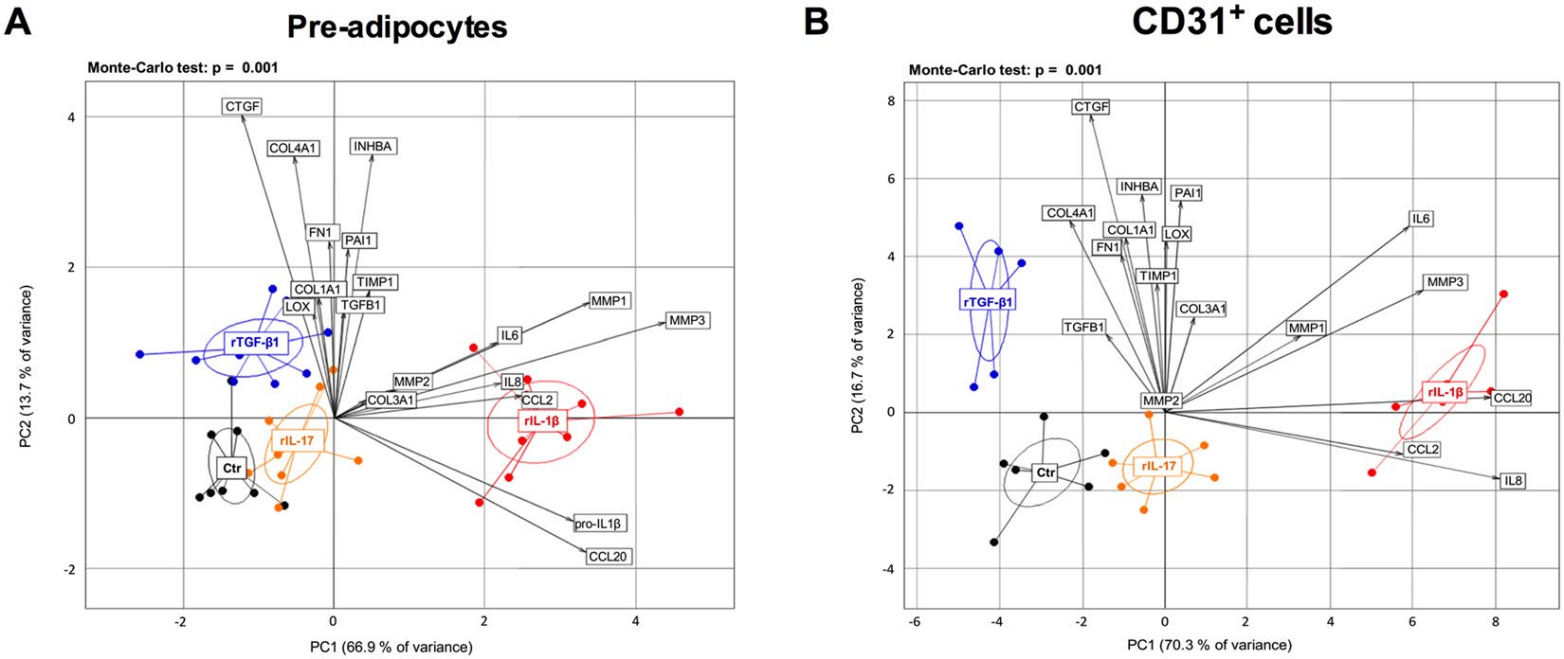
Cytokine-specific effect on fibrotic and inflammatory genes. Principal components analysis (PCA) plots of fibrotic and inflammatory genes in pre-adipocytes (**A**) and CD31^+^ endothelial cells (**B**) treated or not by rIL-17 (10 ng/mL), rIL-1β (10 ng/mL) or rTGF-β1 (5 ng/mL). Points represent individual cell cultures in untreated control (Ctr) or cytokine-treated conditions as indicated. Groups are identified by color and circled with a median elliptical centroid. Arrows depict the contribution of each gene to sample coordinates. P values were obtained by Monte Carlo rank test.

The contribution of IL-1β and IL-17 to adipose tissue fibrotic alterations was explored in pre-adipocytes and CD31^+^ endothelial cells and compared to the effects of TGF-β1 on extracellular matrix components, remodeling enzymes and pro-fibrotic factors. To illustrate an integrated scheme of cytokine and pathway specific regulation, we used principal component analysis (PCA) of 18 fibrogenesis- and inflammation-related genes. The expression profile of both cell types was significantly (Monte-Carlo test, p = 0.001) affected by recombinant cytokines and rTGF-β1 (Fig. 3A,B). The first principal component axis (PC1) contained 67 and 70% of the total inertia in pre-adipocytes and CD31^+^ endothelial cells, respectively. Inflammation-related genes were the main contributors to PC1, while PC2 (14 to 17% of variance) was mainly constituted by fibrosis-associated genes. This analysis highlights that inflammation-related and fibrosis-related genes separate respectively rIL-1β and rTGF-β1 treated cells from controls. Otherwise, cells treated by rIL-17 were more similar to untreated cells on both PC1 and PC2 axes. The data illustrate that the pro-inflammatory effects of IL-1β and IL-17 were not associated with a pro-fibrotic effect.

### Down-regulation of metabolic genes in primary human adipocytes

**Figure 4:**
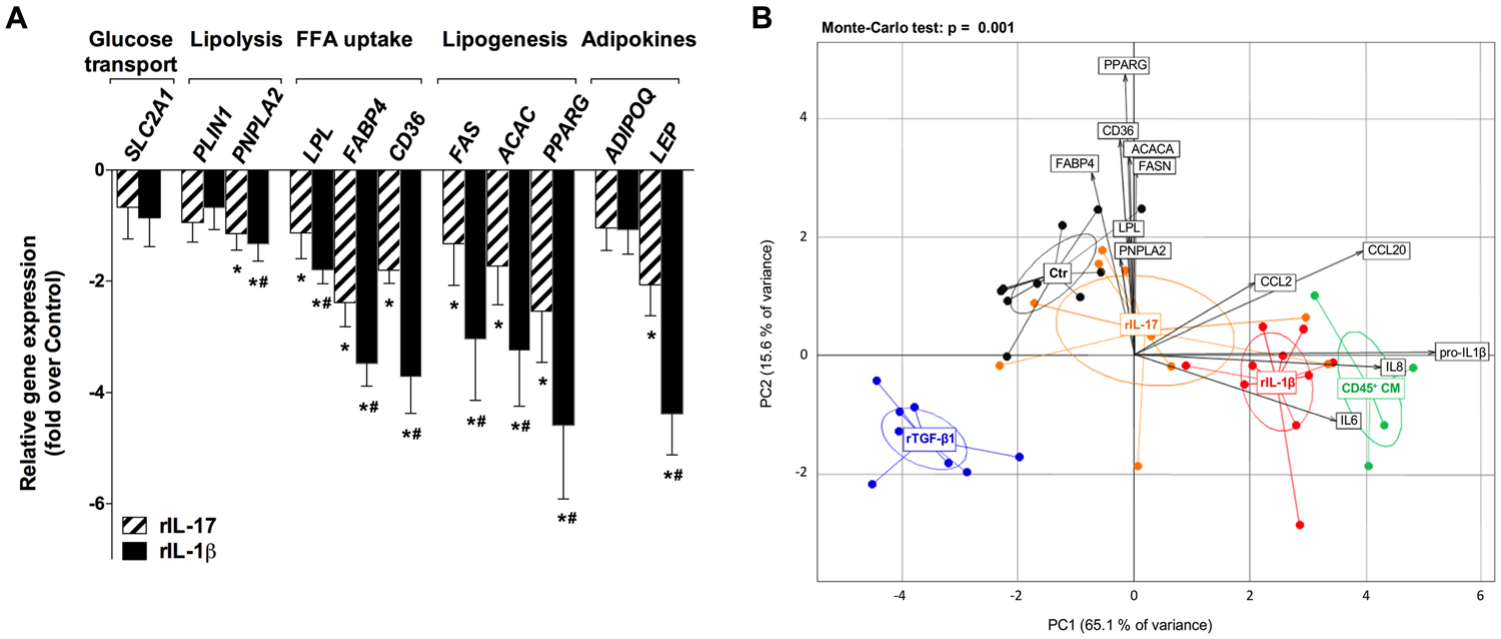
Down-regulation of metabolic genes in primary human adipocytes. (**A**) Metabolic gene mRNA levels were analyzed by QuantiGene Plex in primary human adipocytes cultured in Puramatrix hydrogel for 72 h (n = 10) and treated or not by rIL-17 (10 ng/mL) or rIL-1β (10 ng/mL). *P < 0.05 *versus* untreated cells. ^#^ *P* < 0.05 *versus* rIL-17 treated cells. (**B**) Principal components analysis (PCA) plots of metabolic and inflammatory genes in adipocytes. Points represent individual adipocyte 3D culture in untreated control (Ctr) condition or in presence of rIL-17 (10 ng/mL), rIL-1β (10 ng/mL), rTGF-β1 (5 ng/mL) or CD45^+^ CM as indicated. Groups are identified by color and circled with a median elliptical centroid. Arrows depict the contribution of each gene to sample coordinates. P value was obtained by Monte Carlo rank test.

The effects of recombinant cytokines on genes involved in metabolic and secretory functions of adipocytes were investigated in human primary adipocytes cultured in a 3D setting. We found that rIL-17 and prominently rIL-1β exerted catabolic effects witnessed by down regulation of a series of genes related to lipolysis (*PNPLA2*), *de novo* lipogenesis (*ACACA*, *FASN*, *PPARG*), fatty acid uptake (*LPL*, *CD36*, *FABP4*) and adipokine (*LEP*). Other genes, including *SLC2A1*, *PLIN1* and *ADIPOQ* were not significantly reduced (Fig. 4A).

We extended this metabolic exploration to CD45^+^ cell conditioned media (CD45^+^ CM) from obese omental adipose tissue to address the effects of immune cell-derived cytokines in the context of adipose tissue microenvironment. Additionally, a potential implication of TGF-β1 to alter adipocyte metabolic functions was tested. PCA analysis of inflammatory (PC1) and metabolic (PC2) genes significantly clusterized primary human adipocytes upon treatment (Fig. 4B). Cells treated with rIL-1β or CD45^+^ CM were separated according to both PC1 (65% of variance) and PC2 (15% of variance), with contribution of inflammatory genes in the positive direction on PC1 and metabolic genes in the negative direction on PC2. Cells treated with rIL-17 followed the same pattern, but were closer to the control group on both axes. These data indicate that both cytokines exert coincident catabolic and pro-inflammatory effects in human adipocytes. By contrast, adipocyte response to rTGF-β1 was characterized by a catabolic gene expression profile on PC2 associated with an anti-inflammatory profile on PC1.

### Contribution of IL-1β and IL-17 to inflammatory and metabolic responses

**Figure 5:**
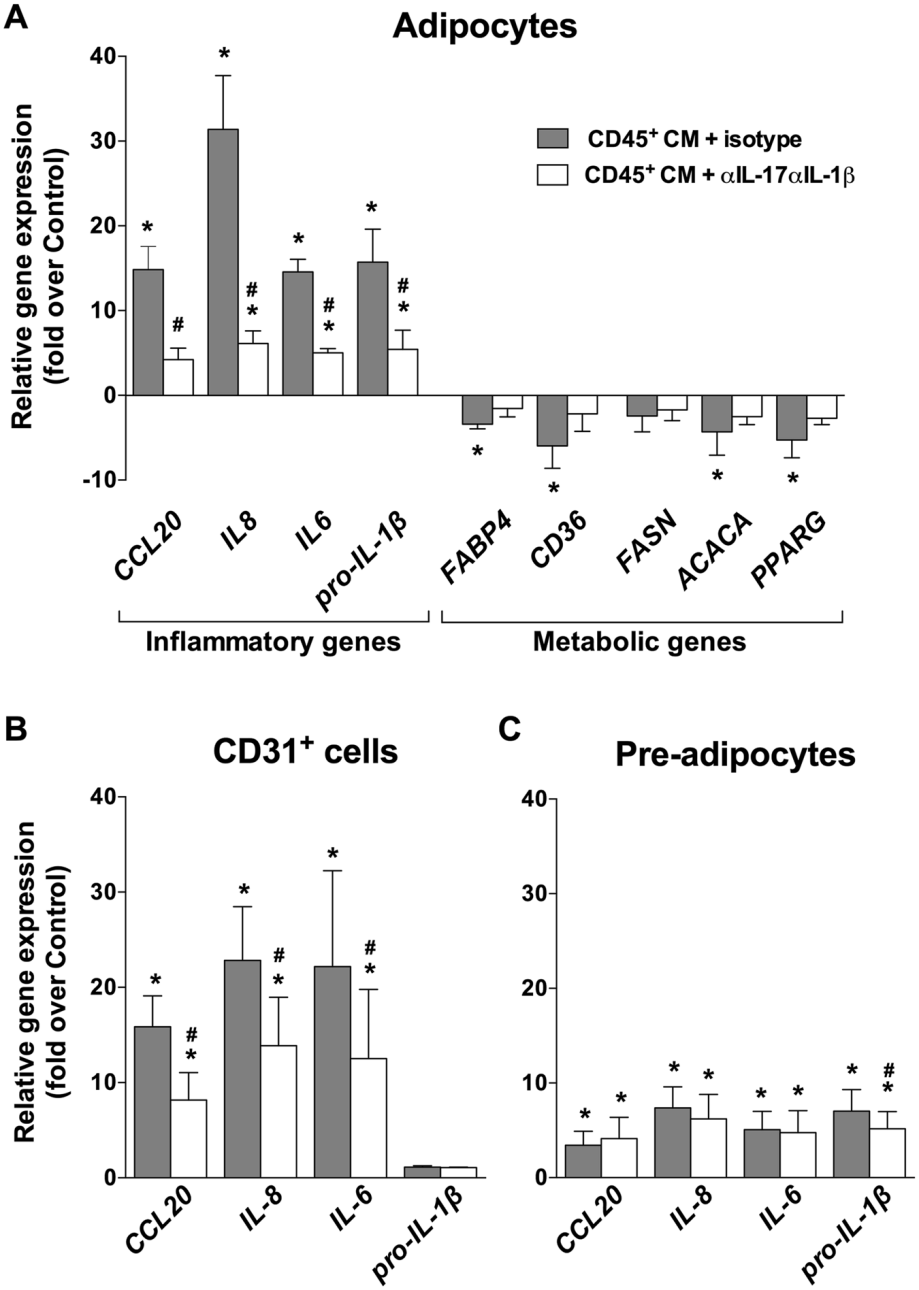
Cell-specific contribution of IL-1β and IL-17 to inflammatory and metabolic gene regulation. (**A**) Metabolic and inflammatory genes were quantified by QuantiGene Plex in adipocytes (n = 4). Inflammatory gene levels were analyzed by RT-qPCR in (**B**) CD31^+^ endothelial cells (n = 6) and (**C**) pre-adipocytes (n = 6). Cells were cultured in presence of CD45^+^ cells conditioned medium (CD45^+^ CM), with addition of isotype control (grey bars) or anti-IL-17 (αIL-17) and anti-IL-1β (αIL-1β) neutralizing (white bars) antibodies. *P < 0.05 *versus* untreated cells. ^#^ *P* < 0.05 *versus* CD45^+^ CM + isotype treated cells. Data are shown as mean ± SEM.

To evaluate the relative contribution of IL-1β and IL-17 among other immune cell-derived secreted factors, the effects of CD45^+^ CM were assessed in presence or not of IL-1β and IL-17 neutralizing antibodies. In adipocytes, antibody-mediated neutralization of these cytokines markedly dampened inflammatory responses, as shown by a potent inhibitory effect (− 65 to − 80%) on inflammatory gene expression (Fig. 5A). These data identify IL-1β and IL-17 as major immune cell-derived factors driving inflammatory alterations in human adipocytes. CD45^+^ CM-induced down-regulation of metabolic genes was also reversed in presence of anti-IL-1β and anti-IL-17 antibodies, although to a lesser extent (− 30 to − 60%) than inflammatory gene overexpression (Fig. 5A). CD45^+^ CM promoted a strong inflammatory response in CD31^+^ cells, which was potently inhibited (up to − 49%) in presence of IL-1β and IL-17 neutralizing antibodies (Fig. 5B). In pre-adipocytes, however, inflammatory gene overexpression was less pronounced and virtually not affected by IL-1β and IL-17 neutralization (Fig. 5C). These data show that human adipose tissue non-immune cells are differentially responsive to the inflammatory effects of immune cell-derived cytokines/factors, including IL-1β and IL-17.

## Discussion

Adipose tissue is composed of a variety of structural, metabolic and immune cells, which communicate through a network of mediators. In obesity, accumulation of immune cells compromises local cytokine production, with deleterious consequences on adipose tissue biology. Complex cross-talks, potentially involving a number of paracrine factors and cell types, are not precisely understood, especially in the human adipose tissue.

We previously identified macrophage-derived IL-1β and Th17 cytokines as major contributors of a chronic inflammatory state in the adipose tissue of obese subjects^25^. We showed here that increased release of IL-1β by omental adipose tissue of obese subjects contributed to promote IL-17 secretion by blood memory T cells, supporting a close interplay between the two cytokines. Moreover, we extended their deleterious implication in human adipose tissue by showing that they induce pro-inflammatory and catabolic responses in non-immune cells. Although IL-17 contributes to these cellular alterations, our data point towards a markedly more potent effect of IL-1β, adding further support for its role of “master” regulator of adipose tissue dysfunctional state in human obesity.

In response to these immune cell-derived cytokines, pre-adipocytes, endothelial cells and mature adipocytes undergo marked inflammatory activation, promoting enhanced production of chemokines known to attract and maintain immune cells within adipose tissue. As such, non-immune cells could amplify the signals required for recruitment of macrophage precursors (CCL2), neutrophils (IL-8) or T cells (CCL20). Our data indicate that IL-1β and IL-17 are important players in this vicious circle. However, adipose non-immune cells might contribute differentially to the release of inflammation-related factors in response to these cytokines, as suggested by their distinct secretory capacity *in vitro*. Our data support a key role for adipocytes, given the high level of inflammatory factors that they release *in vitro*. Moreover, the potent inhibitory effect of IL-1β and IL-17 neutralization on CD45^+^ CM-induced inflammation identifies adipocytes as major targets of the pro-inflammatory effect of both cytokines in obese adipose tissue.

*Pro-IL-1β* was one of the most up-regulated genes by IL-1β it-self, in line with the well established self-amplification of this cytokine in different cellular models^35, 36^. Interestingly, rIL-17 induced *pro-IL-1β* gene in adipocytes and increased the release of low amounts of the mature cytokine. This extended the cellular targets of the paracrine dialogue mediated by Th17 cytokines and IL-1β in human adipose tissue. Further studies are needed to substantiate the molecular mechanisms, by which IL-17 activates the NLRP3/caspase1 dependent pathways driving pro-IL-1β maturation and release^37, 38^ in human adipocytes. Of note, IL-17 was shown to enhance NLRP3-dependent IL-1β release by human keratinocytes^39^, suggesting that the cytokine has the capacity to promote IL-1β production by non-immune cells.

In obesity, chronic inflammation associates with accumulation of several fibrosis components in the adipose tissue. Our team has highlighted the presence of collagen types I and III in fibrotic areas and stressed the leading roles of pre-adipocytes and endothelial cells in human adipose tissue fibrogenesis^10, 40^. Recently, we proposed the contribution of basement membrane components such as collagen IV in adipose tissue fibrosis^34^. Our current data confirmed rTGF-β1-mediated up-regulation of fibrogenic genes^10, 34^, but failed to demonstrate significant fibrotic activation in response to rIL-17 or rIL-1β. Since both cytokines have been implicated in mediating experimental pulmonary fibrosis in mice^41, 42^, their fibrogenic potential might be cell-specific and/or dependent on tissue local inflammatory milieu.

Within the adipose tissue, adipocytes are exposed to the effect of numerous factors, including cytokines, metabolites, hormones and even pollutants, which affect their metabolic capacity and cellular functions. Here, we enlighten a role for IL-1β, IL-17 and TGF-β1 to induce a catabolic state in these cells. rIL-1β catabolic and inflammatory effects were both of higher magnitude than those produced by rIL-17. These observations support potential interactions between inflammatory and metabolic pathways in human adipocytes. By contrast, however, rTGF-β1 exerted a catabolic effect associated with an anti-inflammatory response of these cells. Thus, cytokine-specific impact on adipocyte metabolic capacity might be driven in relation (IL-1β, IL-17) or independently (TGF-β1) of inflammation.

Although CD45^+^ CM exerted a potent catabolic effect in adipocytes, this effect was only partly blocked by IL-1β and IL-17 neutralization in sharp contrast with the powerful effect of the antibodies to dampen inflammatory responses. These data suggest a key role for both IL-1β and IL-17 to promote inflammation in adipocytes, while additional factors might contribute to metabolic deregulation in the context of adipose tissue microenvironment. Among numerous factors produced by human adipose tissue CD45^+^ cells, IL-6 and/or TNF-α are likely candidates, given their well established role to promote inflammation and insulin resistance in adipocytes^14^. Additionally, our current data highlight marked down-regulation of metabolic gene expression in response to TGF-β1, suggesting the involvement of this factor in the catabolic effects of CD45^+^ CM. The hierarchy and kinetic between these cytokines and/or additional paracrine factors in perturbing adipocyte metabolism remains to be fully deciphered.

One limitation of this *in vitro* study relies on the lack of IL-22 receptor (*IL-22RA1*) gene expression in human adipose tissue cell models. As previously reported in human keratinocytes^43^, *IL-22RA1* expression could be lost in our culture conditions. Thus, further studies are needed to decipher the proper implication of IL-22 in human adipose tissue pathology.

Collectively, these data identify a role for IL-17 to promote inflammatory and catabolic responses in adipose tissue non-immune cells. Although concordant with those elicited by IL-1β, cell responses to IL-17 were less pronounced in all experimental setting. We conclude that Th17 cell-derived cytokines participate in the deleterious processes leading to adipose tissue dysfunction in obesity, albeit more modestly than IL-1β. Coupled with our previous data, we propose a scenario, in which the pathogenicity of IL-1β and Th17 cytokines relies on amplification of their release through macrophage-T cell interactions, associated with their concordant capacity to induce pro-inflammatory and catabolic responses in adipose tissue non-immune cells. Our data further enlighten cytokine-specific actions on distinct aspects of adipose tissue deterioration. IL-17, IL-1β and TGF-β1 all three impacted on the metabolic capacity of adipocytes, but exerted clearly distinct effects on inflammation and fibrogenesis. This suggests that complex interplays between immune and non-immune cells drive obesity-induced cellular alterations in adipose tissue. Identifying the whole spectrum of cytokine specific actions on non immune cells will be required to propose new ways to tame adipose tissue metabolic deterioration in human obesity.

## Methods

The study was conducted in accordance with the Helsinki Declaration and approved by the Ethics Committee (CPP Ile-de-France 1). All obese subjects provided written informed consent when included in the surgery program (Fibrota NCT01655017).

### Adipose tissue explants

Omental adipose tissue biopsies were obtained in 8 severely obese subjects (F/M 4/4; age 52.2 ± 3.2 years; BMI 42.2 ± 1.9 kg/m²) during gastric sleeve or bypass and in 3 non obese controls during elective surgery for hernia. Biopsies were minced and 0.1 g was incubated in 1 mL of endothelial cell basal medium (Promocell, Heidelberg, Germany) containing 1% Fetal Bovine Serum and 1% penicillin-streptomycin for 24 h. Omental adipose tissue conditioned media (omCM) were analyzed for cytokine releases using Luminex technology (HTH17MAG-14K; Millipore, Billerica, MA, USA).

### Blood memory T cells

Whole mononuclear cells were recovered from human blood by Ficoll-Hypaque (GE Healthcare, Little Chalfont, UK) gradient centrifugation. Total cells were stained for fluorescence-activated cell sorting (FACS) with CD4-BV421 (562424; BD Biosciences, San Jose, CA, USA), CD25-FITC (555431; BD Biosciences), CD62L-PE (12-0629-42; Affymetrix, Santa Clara, CA, USA) and CD45RA-PE-Cy7 (60-0458-T100; Tonbo Biosciences, San Diego, CA, USA). Blood memory T cells (CD4^+^CD25^-^CD62L^+^CD45RA^-^) were isolated by sorting with a FACS Aria (BD Biosciences) and cultured for 7 days at 1.10^6^ cells per mL in X-Vivo + 2% Fetal Bovine Serum, with plate-bound anti-CD3 (2.5 µg/mL; UCHT1) and soluble anti-CD28 (1 µg/mL; 37407) antibodies (R&D Systems, Minneapolis, MN, USA), with or without omCM (1/4 vol/vol). Blood memory T cells conditioned media were analyzed for cytokines using Luminex technology (HTH17MAG-14K; Millipore). Cells were used for FACS analysis. Intracellular cytokine detection was performed in cells stimulated with phorbol-12-myristate 13-acetate (30 ng/mL; Sigma-Aldrich, St Quentin Fallavier, France) and ionomycin (1 µg/mL; Alexis Biochemicals, San Diego, CA, USA) at 37 °C for 5 h with GolgiStop (BD Biosciences). After permeabilization with Cytofix/Cytoperm solution (BD Biosciences) cells were incubated with PE-labeled anti-IL-17A (12-7178-42; eBioscience, San Diego, CA, USA) and PE-Cy7-labeled anti-IL-22 (25-7229-42; eBioscience) and analyzed on a FACS LSRII flow cytometer (BD Biosciences) using FACS Diva software (BD Biosciences). Data analysis was performed with Flow Jo 9.4 software (Tree Star, Ashland, OR, USA).

### Conditioned media from adipose CD45^+^ cells

Omental adipose tissue biopsies from 8 severely non-diabetic obese women (age 42.2 ± 2.6 years; BMI 43.1 ± 1.6 kg/m²) were dissociated by collagenase to obtain adipocytes and cells of the stromal vascular fraction (SVF). CD45^+^ immune cells were isolated from SVF using positive selection magnetic beads (Miltenyi Biotec, Bergisch Gladbach, Germany) and cultured for 48 h (1.10^6^ cells per mL) in RPMI 1640 (Lonza, Berkshire, U.K.) with 10% Fetal Bovine Serum, 1% penicillin-streptomycin, 1% sodium pyruvate and 1% NEAA to obtain conditioned medium (CD45^+^ CM).

### Adipose non-immune cell isolation and culture

Non-immune cells were isolated from subcutaneous adipose tissue collected from lipoaspirate in 27 non-obese female subjects (age 44.2 ± 3.3 years; BMI 22.3 ± 0.5 kg/m²) and submitted to collagenase digestion. Pre-adipocytes, selected from SVF cells after at least 2 passages to eliminate non pre-adipocyte cell contamination^9^, were cultured (4.10^4^ cells per mL) in DMEM/F12, supplemented with 1% penicillin-streptomycin and human insulin (5 nM). Endothelial cells were isolated from SVF cells by CD31^+^ bead positive selection (Stemcell Technologies, Grenoble, France), grown in ECGM medium (Promocell) as described in^32^ and cultured (4.10^4^ cells per mL) in ECBM (1% penicillin-streptomycin and 1% Bovine Serum Albumin fatty acid free). Floating adipocytes (1.10^5^ cells per mL) were incorporated in hydrogel (Puramatrix, Corning, Bedford, MA, USA) for 3D setting according to the protocol described in^33^.

Cells were cultured without or with human recombinant cytokines, rIL-1β, rIL-17 (10 ng/mL; Miltenyi Biotec) or rTGF-β1 (5 ng/mL; Bio-Techne, Minneapolis, MN, USA) for 72–96 h as indicated, with medium replacement after 48 h. For some experiments, CD45^+^ CM was added (1/8 vol/vol) to cell culture in presence of isotype control (mIgG1κ, 2.55 µg/mL) or anti-IL-1β (2.5 µg/mL) and anti-IL-17 (50 ng/mL) neutralizing antibodies (eBioscience). By the end of the culture periods, media were recovered and kept frozen at −80 °C until use. Pre-adipocytes and endothelial cells were harvested in RLT Qiagen buffer + 1% β-mercaptoethanol. Adipocytes were immediately lysed using QuantiGene Sample Processing kit (Affymetrix).

### Gene expression analysis and secretion

Gene expression analysis in pre-adipocytes and CD31^+^ cells was performed by PCR Array (Human Fibrosis PCR Array, Qiagen, Courtaboeuf, France) or RT-PCR. Total RNA was extracted using the RNeasy Mini Kit (Qiagen). cDNAs were synthesized from and prepared with M-MLV reverse transcriptase (Promega, Fitchburg, WI, USA) and Supercript II reverse transcriptase (Life Technologies, Carlsbad, CA, USA). Exiqon primers were used for quantitative Real-Time PCR using the 7300 real-time PCR system (Applied Biosystem, Foster City, CA, USA). Data were normalized according to the RPLP0 gene expression. In 3D cultured adipocytes, analysis of gene expression was performed with QuantiGene 2.0 Plex Assay kit (Affymetrix) according to manufacturer instructions. Plates were analyzed in the Bio-Plex Luminex 200 system (BioRad, Hercules, CA; USA), gene expression was calculated using Bio-Plex Manager 5.0 software, and results were normalized to 18 S expression. Concentrations of secreted factors were assessed in the three non-immune cell type culture media by the Luminex technology (Millipore).

### Statistical analyses

All data are shown as mean ± SEM. Differences between groups were assessed by paired or unpaired non parametric tests, except for Fig. 4B (paired Student’s t test). Correlations were assessed by Spearman’s test. A *P* value < 0.05 was considered statistically significant. Statistical analyses were done with GraphPad Prism version 6.0 (GraphPad Software). Principal component analysis (PCA) was performed using the R 3.1.2 program and ade4 package. Interclass PCA were computed and statistically assessed by a Monte Carlo rank test.

## Electronic supplementary material

Dataset 1
