# Supplementary material for: Immune cell-derived cytokines contribute to obesity-related inflammation, fibrogenesis and metabolic deregulation in human adipose tissue
Source: Sci Rep. 2017 Jun 7;7:3000. doi: 10.1038/s41598-017-02660-w (PMC5462798; doi:10.1038/s41598-017-02660-w)
Supplement: Supplementary file 1 — Dataset 1 [file 41598_2017_2660_MOESM1_ESM.doc]

Immune cell-derived cytokines contribute to obesity-related inflammation, fibrogenesis and metabolic deregulation in human adipose tissue.

Charles Caër^1,2,3 $^, Christine Rouault^1,2,3 $^, Tiphaine Le Roy^1,2,3^, Christine Poitou^1,2,3,4^, Judith Aron-Wisnewsky^1,2,3,4^, Adriana Torcivia^5^, Jean-Christophe Bichet^6^, Karine Clément^1,2,3,4^, Michèle Guerre-Millo^1,2,3*^ and Sébastien André^1,2,3^

^1^ INSERM, UMR_S 1166, Team 6 Nutriomics, F-75013, Paris, France;

^2^ Sorbonne Universités, UPMC Université Paris 06, UMR_S 1166, F-75005, Paris, France;

^3^ Institute of Cardiometabolism and Nutrition, ICAN, Pitié-Salpêtrière Hospital, Assistance Publique Hôpitaux de Paris, F-75013, Paris, France;

^4^ Assistance Publique Hôpitaux de Paris, Pitié-Salpêtrière Hospital, Nutrition and Endocrinology Department, F-75013, Paris, France;

^5^ Assistance Publique Hôpitaux de Paris, Pitié-Salpêtrière Hospital, Hepato-biliary and Digestive Surgery Department, F-75013, Paris, France;

^6^ Assistance Publique Hôpitaux de Paris, Pitié-Salpêtrière Hospital, Plastic surgery and Mammary Cancer Department, F-75013, Paris, France.

| Cytokines | Cytokine concentration (pg/mL) in omCM | | Correlation with IL-17 release by blood memory T cells | |
| --- | --- | --- | --- | --- |
|  | Lean subjects  (n = 3) | Obese subjects  (n = 8) | r | P |
| IL-6 | 585.8 | 49 377.3 | 0.86 | 0.001 |
| CCL20 | 12.1 | 2 562.2 | 0.87 | 0.001 |
| TNF-α | 1.6 | 68.1 | 0.75 | 0.010 |
| IL-1β | 1.3 | 61.5 | 0.82 | 0.003 |
| IL-10 | 1.2 | 50.2 | 0.88 | 0.001 |
| IL-33 | 35.2 | 9.5 | 0.18 | 0.580 |
| IL-21 | 1.4 | 7.6 | 0.65 | 0.030 |

Supplementary Table S1: Cytokine concentrations in omental adipose tissue conditioned media (omCM) in lean and obese subjects were measured by Luminex. Correlations between cytokine concentration and the rate of IL-17 release in response to omCM in blood memory T cells were assessed by Spearman’s test
